# Clinical evaluation of short 6-mm implants alone, short 8-mm implants combined with osteotome sinus floor elevation and standard 10-mm implants combined with osteotome sinus floor elevation in posterior maxillae: study protocol for a randomized controlled trial

**DOI:** 10.1186/s13063-015-0853-4

**Published:** 2015-07-30

**Authors:** Jun-Yu Shi, Ying-Xin Gu, Shi-Chong Qiao, Long-Fei Zhuang, Xiao-Meng Zhang, Hong-Chang Lai

**Affiliations:** Department of Oral and Maxillofacial Implantology, Shanghai Ninth People’s Hospital, School of Medicine, Shanghai Jiao Tong University, 639 Zhizaoju Road, Shanghai, 200011 China

**Keywords:** Dental implants, Short implants, Osteotome sinus floor elevation, Clinical evaluation

## Abstract

**Background:**

Nowadays, short dental implants are being increasingly applied in extremely resorbed posterior regions. The recent studies have indicated that short implants present a similar success rate to conventional implants. It is assumed that short implants can avoid additional surgical morbidity and are less technically demanding. However, high-quality evidence (≥Ib: evidence from at least one randomized controlled trial) on comparing the clinical outcome of short implants and longer implants combined with osteotome sinus floor elevation (OSFE) technique is limited.

**Methods/Design:**

The proposed study is designed as a prospective single-center, three-arm parallel group, randomized controlled trial. We plan to enroll 150 patients in need of dental implant treatment in the posterior maxilla. The inclusion criteria include: age ≧18 years, partial edentulism in the posterior maxilla for at least 3 months from tooth loss, residual bone height ranging from 6 to 8 mm, sufficient bone width (≥6 mm) in the edentulous region. The patients will be divided into three groups according to a table of random numbers: group 1: short implants (6 mm) alone; group 2: short implants (8 mm) combined with osteotome sinus floor elevation (OSFE); group 3: standard implants (10 mm) combined with OSFE. The assignment will be concealed from the clinical operators until the beginning of implant surgery. The outcome examiners and patients will be kept blinded to the assignment. Implant survival rates, implant success rates, complications, resonance frequency analysis (RFA) measurements, marginal bone level, treatment time and patient-reported outcome (visual analogue scale for intraoperative discomfort and postoperative pain) will be recorded. Clinical re-evaluations will be performed at 12, 24, 36 and 60 months after crown placement.

**Discussion:**

The results of the trial will support better decision-making for dental implant treatment in atrophic maxillary ridges. If favorable, the use of short implants may avoid adjunct procedures used for implant insertion, thus reducing operative time, complexity and postoperative discomfort.

**Trial registration:**

Clinicaltrials.gov identifier: NCT02350075 (registered on 17 February 2015).

## Background

Implant treatment in the posterior maxilla is commonly faced with great challenges due to the limited residual bone height (RBH) and poor bone quality [1]. A cone-beam computed tomographic analysis study based on 199 sinuses showed that RBH ≤4 mm was observed in 46.9 % and 48.9 % of edentulous first and second molar sites [2]. Efforts have been made to allow successful implant treatment in atrophic posterior maxillae. According to current evidence, osteotome sinus floor elevation (OSFE) has been proven to be a predictable surgical procedure with a high success rate to vertically increase the bone volume in atrophic maxilla when the RBH ranges from 6 to 8 mm [3]. However, the additional surgical procedure is technically demanding, and may increase postoperative morbidity and the total cost [4].

As an alternative, short implants are being increasingly applied in extremely resorbed posterior regions. The definitions of short implants varied in the literature. Authors defined implant length less than 11 mm [5], 10 mm [6] or 8 mm [7] as short implants. Telleman argued that because an implant could be placed at a different horizontal level, a short implant should be defined as an implant with a designed intrabony length of 8 mm or less [8].

A recent meta-analysis has indicated that short implants present a similar success rate to conventional implants and the implant length is no longer considered a crucial factor in influencing implant success [9]. However, contradictory results are reported when short implants are inserted in the posterior maxilla. A retrospective cohort study of 4591 Straumann (Standard, Standard Plus, Tapered Effect and Bone Level) implants with up to 10-year follow-up showed that short 6-mm implants in the mandibular posterior sites had a high survival rate of 100 %, while in maxillary posterior positions a survival rate of only 87 % was achieved [10]. On the other hand, several studies reported an acceptable clinical outcome (survival rates range from 90.0 % to 98.3 %) using short implants (5 mm, 6 mm and 7 mm) in the treatment of the posterior maxilla with up to 5–10 years follow-up [11, 12].

To our knowledge, three randomized controlled clinical trials compared short implants versus longer implants in combination with OSFE procedures in the posterior maxilla [13–15]. Similar survival rates were reported in short and longer implant groups after a 1-year follow-up. However, the limited sample size (101 patients in Thoma et al. 2015 [14], 41 patients in Gujie et al. 2014 [13] and 30 patients in Esposito et al. 2014 [15]) might lead to insufficient statistical power. In addition, the lateral sinus floor elevation technique was performed in those patients with RBH from 5 to 8 mm, while the OSFE technique might be more suitable from cost-effectiveness considerations. Thus, there is still an urgent need for randomized controlled clinical trials with long-term data and a sufficient sample size to provide more clinical evidence to decide the best treatment options.

Based on these, we are now designing a randomized controlled trial study with a 5-year follow-up trying to answer the research question: which is the best treatment option in atrophic maxilla with RBH from 6 to 8 mm: short 6-mm implants alone or short 8-mm implants combined with OSFE or standard 10-mm implants combined with OSFE?

### Objective and hypotheses

The aim of the study is to evaluate the clinical success and the patient-reported outcome of intraoperative and postoperative pain of short implants (6 mm) alone, short implants (8 mm) combined with OSFE and standard implants (10 mm) combined with OSFE for treating atrophic posterior maxillary ridges of partially edentulous adults. Considered outcomes include implant survival rates, complications, resonance frequency analysis (RFA) measurements, marginal bone level, treatment time and patient-reported outcome (visual analogue scale for (VAS) intraoperative discomfort and postoperative pain)

## Method/Design

### Overview

The proposed study is designed as a prospective single-center, three-arm parallel group, randomized controlled trial. We plan to enroll 225 patients in need of dental implant treatment in the posterior maxilla. The study (Number [2015]16) has been approved by the Ethics Committee of Shanghai Ninth People’s Hospital, China. In addition, the study has been registered in ClinicalTrials.gov and the identifier number is NCT02350075. The clinical component of the study will be initiated in September 2015 at the Department of Oral and Maxillofacial Implantology, Shanghai Ninth People’s Hospital, Shanghai Jiao Tong University, China.

The systematic health condition of all the participants will be recorded and they should fulfill the following criteria. Before the implant surgery, all patients will receive clinical and radiographic assessment. All patients will be required to receive oral hygiene instructions before implant surgery. Panoramic and periapical radiographs will be taken to assess the initial bone height and width. If necessary, cone-beam computed tomography will be performed.

### Inclusion criteria

- Aged over 18 years old
- Partial edentulism (all incisors and canines present) for at least 3 months from tooth loss
- Residual bone height ranging from 6 to 8 mm
- Sufficient bone width in the edentulous region (≥6 mm)

### Exclusion criteria

- Medically compromised patients (American Society of Anesthesiologists (ASA) classification III–IV)
- Heavy smokers (>10 cigarettes/day)
- Uncontrolled diabetes mellitus
- Complete edentulism

### Recruitment

Recruitment will be performed in the Department of Oral and Maxillofacial Implantology, Shanghai Ninth People’s Hospital. Eligible patients will receive the study information and consent forms. No patients will be included in the study until the consent forms are signed.

### Allocation and blinding

The patients will be divided into three groups using randomization tables allocating the patient a number within a corresponding envelope: group 1: short implants (6 mm) alone; group 2: short implants (8 mm) combined with OSFE; group 3: standard implants (10 mm) combined with OSFE. The assignment will be concealed from the clinical operators until the beginning of implant surgery. The outcome examiners and patients will be kept blinded to the assignment.

### Surgical procedures

For all the cases, Straumann (Institute Straumann AG, Basel, Switzerland) Standard Plus implants will be placed. Following a mid-crestal incision, a full-thickness mucoperiosteal flap will be reflected. In group 1, 6-mm implants will be inserted according to the manufacturer’s instructions. In group 2, after implant, the site will prepared to a depth of about 1–2 mm away from the sinus floor, a modification of Summers’ OSFE will be performed to elevate the sinus membrane to a depth of 8 mm. In group 3, similar procedures will be performed until the sinus membrane is elevated to a depth of 10 mm. The OSFE procedure has been described in detail previously [16]. After surgery, anti-inflammatory amoxicillin (Xinya Co., Shanghai, China, 500 mg, three times a day for 7 days) and metronidazole (Xinyi Wanxiang, Shanghai, China, 400 mg, three times a day for 7 days) will be used. In addition, chlorhexidine oral rinse (0.12 %) will be prescribed for 60 s two times a day for 14 days. A one-stage protocol will be performed. After a healing time of 3–4 months, an impression will be taken at the implant level. Straumann synOcta titanium abutments will be fixed onto the implants. Porcelain (Ivoclar Vivadent AG, Schaan, Liechtenstein) fused to noble metal (Heraeus Kulzer Corporation, Hanau, Germany) crowns and bridges will be fabricated.

### Outcomes

#### Baseline assessment

Resonance frequency analysis (RFA) measurements, treatment time and patient-reported outcome will be recorded. RFA assessment will be performed immediately after surgery, 2 weeks later and 3 months after surgery. RFA measurements will be conducted by the Osstell™ mentor (Integration Diagnostics AB, Göteborg, Sweden). Treatment time will be calculated from incision beginning to suture finishing. Patients will be asked to give their answers regarding intraoperative discomfort and postoperative pain immediately and two weeks after surgery using a 100-mm visual analogue scale (VAS) with word descriptors "very dissatisfied" to "very satisfied" on the left and right respectively.

#### Follow-up assessment

All the included patients will be recalled for clinical and radiographic assessment at 12, 24, 36, and 60 months after crown placement. Survival rates, complication rates and marginal bone level will be recorded. Implant survival rates are defined as the percentage of implants still retained in the mouth. Implant success is defined according to Cochran et al. 2002 [17]. Both biological and mechanical complications will be recorded. Periapical radiographs will be taken immediately after implant surgery, and at the 1-year, 3-year, and 5-year examination. For standardization, a paralleling technique with a Rinn film holder (XCP Instruments; Rinn Corporation, Elgin, IL, USA) will be conducted. Radiographic analysis will be conducted by a software program (SIDEXIS 1.12; Sirona Dental Systems GmbH, Bensheim, Germany). Biological complications refer to peri-implant mucositis (bleeding on probing positive, and without surrounding bone loss) and peri-implantitis (bleeding on probing positive, probe pocket depth ≥5 mm and with surrounding bone loss), and technical complications are veneer ceramic chipping, framework fracture, loss of retention, screw loosening, abutment and implant fracture.

#### Sample size

The primary outcome is the implant survival rate. The criterion for significance is set at α = 0.05 (type I error) and at β = 0.10 (type II error). The analysis is two-tailed. The survival rate reported by Esposito et al. 2014 (92 % in the longer group and 85.7 % in the short group) is used to estimate the sample size [15]. Forty-two cases per group can result in a power of 80 %. Assuming the dropout rate at 20 %, 50 cases per group and 150 cases in total are finally determined.

#### Statistical analysis

Generalized estimating equations will be used to manage the correlated relationships between multiple implants in the same patient. The data of different patients are assumed to be independent. Each patient will be assigned a number, which will be an indicator (X) for primary and secondary outcome (Y). For the patient (i) and implants (j), mean model is logit(E(Yij|Xij)) = beta0 + beta1*I(Xij = 2) + beta2*I(Xij = 3), and variance model is Var(Yij|Xij) = E(Yij|Xij)*(1-E(Yij|Xij)). For patient-reported outcome (VAS), ANOVA will be used to assess the difference among the three groups. The level of significance is set at 0.05.

#### Missing data

Sample size calculation was performed accounting for possible loss to follow-up. Moreover, we will account for the data not missing at random due to unbalanced loss to follow-up by handling drop-outs as nonsuccess or nonsurvival using the intention-to-treat principle.

### Ethical considerations

#### Ethical approval

The study has been approved by the Ethics Committee of Shanghai Ninth People’s Hospital, China (Number [2015]16). Eligible patients will receive the study information and consent forms. No patients will be included in the study until the consent forms are signed.

#### Withdrawal

Patients will be informed that they have the right to withdraw from the study at any time without giving reasons. Regardless of withdrawal, patients will be provided with any treatment requested by them.

#### Dissemination of results

The results of the study will be published in international peer-reviewed journals. A summary of the study results will also be saved at Clinicaltrials.gov to allow general access to obtain findings.

## Discussion

Implant treatment in the posterior maxilla is commonly faced with great challenges due to the limited residual bone height and poor bone quality. In 2013, our study demonstrated that high survival rates can be achieved after 5–10years for Straumann SLA Standard Plus short implants (6 or 8 mm) in the posterior region, without severe marginal bone loss and complications [18]. This result shows that Straumann short 6-mm implants are predictable and reliable in the posterior maxilla. In addition, our previous studies have also indicated that the OSFE technique is also predictable and reliable in the posterior maxilla [16, 19]. Therefore, our research group is very interested in the long-term treatment outcome of short implants alone and longer implants combined with OSFE.

The results of the trial will support better decision-making for dental implant treatment in atrophic maxillary ridges. If favorable, the use of the short implant may avoid adjunct procedures used for implant insertion, thus reducing operative time, complexity and postoperative discomfort.

### Trial status

The trial was registered at Clinicaltrials.org and the study is open for recruitment.

## Abbreviations

ASA: American Society of Anesthesiologists
OSFE: osteotome sinus floor elevation
RBH: residual bone height
RFA: resonance frequency analysis
VAS: visual analogue scale
